# Microbial Profile and Antimicrobial Susceptibility Pattern in Diabetic Foot Ulcer Patients Attending a Tertiary Care Hospital

**DOI:** 10.7759/cureus.29770

**Published:** 2022-09-30

**Authors:** Shahzad Ahmad, Muhammad Sajjad A Khan, Muhammad H Shah, Aimal Khan, Raheela Bano, Mustafa Qazi

**Affiliations:** 1 Medicine, Northwest General Hospital and Research Center, Peshawar, PAK; 2 Pathology, Gomal Medical College, Peshawar, PAK; 3 Medicine and Surgery, Northwest General Hospital and Research Center, Peshawar, PAK; 4 Medicine and Surgery, Northwest School of Medicine, Peshawar, PAK

**Keywords:** diabetic foot ulcer, foot ulcer, pakistan, antibiotic susceptibility, management, diabetic foot, bacterial profile, diabetes mellitus

## Abstract

Background

This study aimed to assess the bacterial profile of diabetes patients with an infected foot and their antimicrobial susceptibility pattern in a tertiary care hospital.

Methodology

We conducted a six-month prospective study at a hospital in Peshawar, Pakistan. Demographics and clinical characteristics such as age, sex, type and duration of diabetes, glycemic control, presence of retinopathy, nephropathy, neuropathy, peripheral vascular disease, ulcer size, outcomes, and the number of admitted days at the facility were collected. Pus or discharges from the ulcer base and debrided necrotic tissue were obtained. Gram staining was performed on the samples which were isolated on chocolate agar and MacConkey agar. Incubation was done for 24 hours at a temperature of 37°C, and isolates were identified using standard bacteriological methods. The Kirby-Bauer testing method was used to assess antibiotic susceptibility.

Results

A total of 200 patients received a diagnosis of diabetic foot ulcer at the hospital during the study period. The age of the patients ranged from 24 to 92 years, with a mean age of 58.12 years (standard deviation (SD) = 12.494). The mean HbA1c level was 9.33% (SD = 2.050). The mean duration of diabetes mellitus was 12.3 years (SD = 6.181). In total, 96 (66.2%) isolates were gram-negative bacteria, while 49 (33.8%) were gram-positive bacteria. Among the gram-negative bacteria, *Pseudomonas *spp. was the most reported (15.9%), whereas methicillin-resistant *Staphylococcus aureus* was the most reported gram-positive bacteria (20.7%). Amikacin was found to be the most effective (45%) in treating diabetic foot ulcers, followed by tineam and meropenem being equally effective at a susceptibility of 44%. The highest resistance of the microbes was to the drug trimethoprim (44.5%).

Conclusions

The pathogens causing diabetic foot ulcers show sensitivity to many of the routinely used medications. However, resistance is being developed to some of the antibiotics such as trimethoprim. Therefore, the culture of the specimen to identify the causative agent and adequate knowledge of the susceptibility pattern are critical for the appropriate management of diabetic foot ulcers.

## Introduction

Diabetes mellitus (DM) is a serious health concern and a leading cause of morbidity and mortality. It is estimated that every six seconds someone dies due to the consequences of this devastating disorder [1]. In 2015, approximately five million diabetes-related deaths were reported in low-income and middle-income countries [2].

Asia is considered an epicenter of the diabetes epidemic. In 2017, the diabetic population of South Asian countries was 80 million, and the number is projected to reach 151 million by 2045 [3]. According to the International Diabetes Federation (IDF), Pakistan is among the top 10 countries with the highest prevalence of diabetes, with a prevalence of 8.7% reported in the first national survey conducted in 1994 [4]. Surprisingly, the most recent survey of 2016 revealed a 26.3% prevalence among young adults in the country [5].

Improper control of DM predisposes patients to complications such as gangrenous and non-gangrenous ulcers in the feet where there is loss of sensation due to neuropathy, peripheral vascular disease (PAD), and coronary artery disease. Neuropathy, PAD, and pressure overload are considered to be the predictive factors for ulcers [6]. Diabetic foot ulcers are the most frequently occurring infections seen in about 15-25% of diabetics and most frequently affect old patients with poor diabetic control [7]. The worst-case scenario for diabetic foot ulcer patients, who are at a much higher risk, is when the complications lead to the need for limb amputation. This happens to hundreds to thousands of DM patients in a year [8].

Studies on the types of foot infections and the causative organisms in DM patients have revealed that there is usually one microbe causing the condition, with *Staphylococcus aureus* and *Streptococcus *species being the most common pathogens [9-11]. On the other hand, infections with more than one causative pathogen are the ones with the worst prognosis. Among the frequently reported organisms are *Pseudomonas *spp., *Escherichia coli*, and *Klebsiella *spp. [11].

Proper care, implementation of policies and guidelines, patient counseling, and awareness of the patients regarding the potential complication of DM can significantly lower admissions, duration of hospital stay, and the need for amputations. The earlier the lesions are noticed and reported to the physician, the earlier appropriate medications can be started which will lead to a notable change in the clinical outcomes. Very little information on this subject is available in our region (Peshawar, Khyber Pakhtunkhwa (KPK)). Studies need to be conducted to reduce the incidence of diabetic foot ulcers and their associated costs and improve the quality of life of patients.

This study aimed to assess the bacterial profile of diabetic foot patients and the antimicrobial susceptibility pattern in the setting of a tertiary care hospital.

## Materials and methods

Study design and setting

A prospective, descriptive study was conducted for six months at Northwest General Hospital and Research Center (NWGH & RC) which is a tertiary care hospital in Peshawar, Pakistan. The facility receives patients from all parts of KPK and nearby Afghanistan.

Sample size

A total of 280 diabetic patients with diabetic foot ulcers were included in the study. The sample size was calculated using the World Health Organization formula at a 95% confidence interval using a 5% margin of error at a normal standard deviation.

Data collection

A structured format was used to collect information on demographics and clinical characteristics such as age; sex; type and duration of diabetes; glycemic control (HbA1c); the incidence of microvascular and macrovascular complications, such as retinopathy, nephropathy, neuropathy, and peripheral vascular disease; size and age of ulcer; outcomes at the time of discharge; and the number of admitted days at the hospital. A creatinine value of 150 µmol/L or higher along with detection of albumin in urine was considered nephropathy. Peripheral neuropathy was diagnosed from symptoms of numbness, tingling, and loss of sensation in the limbs. Ischemic changes in the skin, pain, weak pulses, and hair loss were considered symptoms of peripheral vascular disease. Patients were assessed for signs of infection, ulcers were identified, and their size was determined and expressed in cm^2^.

Specimen collection and processing

Specimens of purulent discharges and dead tissue were collected from the base of the ulcers and sent to the histopathology laboratory at the earliest so they could be processed as soon as possible. Gram staining was performed on the specimens, and, at the same time, they were also inoculated on chocolate and MacConkey agar to isolate and identify the microbes. Isolates were identified according to the standard bacteriological methods after incubation for 24 hours at a temperature of 37°C [12]. The disc diffusion method of Kirby-Bauer was employed in accordance with the guidelines of the Clinical Labs Standards Institute to determine antibiotic susceptibility.

Exclusion criteria

Patients who reported taking antibiotics for more than 24 hours in the last 72-hour period were excluded from the study.

Data analysis

Data analysis was performed using SPSS version 22 (IBM Corp., Armonk, NY, USA).

## Results

A total of 200 patients who visited the facility during the study period were diagnosed as cases of diabetic foot ulcers. Table 1 shows the demographic and clinical characteristics of the study population. The age of the study participants ranged from 24 to 92 years, with a mean age of 58.12 years (standard deviation (SD) = 12.494). The highest number of patients (48.5%) were in the 41-60-year age group, followed by the age group of more than 60 years with 43% of the patients. The ratio of male-to-female participants was 11:9.

**Table 1:** Demographic and clinical characteristics of the patients. HTN: hypertension; CAD: coronary artery disease; CABG: coronary artery bypass graft surgery; CKD: chronic kidney disease; HCV: hepatitis C virus; CLV: chronic liver disease; TBM: tuberculous meningitis; PAD: peripheral artery disease

Parameter	Number of patients (%)
Age	
<25 years	1 (0.5)
26–40 years	16 (8.0)
41–60 years	97 (48.5)
>60 years	86 (43.0)
Gender	
Male	110 (55.0)
Female	90 (45.0)
Diabetes mellitus	
Type 1	2 (1.0)
Type 2	193 (96.5)
Duration of diabetes mellitus	
5–10 years	40 (20.0)
<5 years	44 (22.0)
>10 years	116 (58.0)
Location of ulcer	
Foot	129 (64.)
Lower limbs	22 (11.0)
Sacrum	7 (3.5)
Abdomen	12 (6.0)
Other	21 (10.5)
Comorbidities	
HTN	91 (45.5)
Ischemic heart disease	8 (4.0)
HTN CAD CABG	15 (7.5)
CKD	7 (3.5)
HCV/CLD	6 (3.0)
HTN/Hypothyroidism	3 (1.5)
HTN/CKD	19 (9.5)
Rheumatoid arthritis	1 (0.5)
TBM, HTN	1 (0.5)
Macrovascular complications	
Cerebrovascular accidents	21 (24.4)
CAD	46 (53.5)
PAD	18 (20.9)
All	1 (1.2)
Microvascular complication	
Retinopathy	7 (5.1)
Nephropathy	28 (20.3)
Neuropathy	63 (45.7)
All	40 (29.0)

The mean HbA1c value was 9.33% (SD = 2.050), with the minimum and maximum values being 5% and 15%, respectively. The mean duration of DM was 12.3 years (SD = 6.181). The sites most frequently ulcerated were the foot (64.5%) and limb regions (11%). Hypertension was present in several study participants (45.5%). The most commonly associated microvascular and macrovascular complications were neuropathy (45.7%) and coronary artery diseases (53.5%). In total, 40 (29%) patients had multiple microvascular complications, whereas only one (1.2%) patient had multiple macrovascular complications.

Bacterial isolates from the culture of the ulcers are presented in Table 2. Of the total cultures isolated, 96 (66.2%) were detected to be gram-negative bacteria, while 49 (33.8%) were gram-positive bacteria. Among the gram-negative bacteria, *Pseudomonas *spp. was detected in most of the cultures (15.9%), followed by extended-spectrum beta-lactamase (ESBL) producing *Escherichia coli* (14.5%). On the other hand, methicillin-resistant *Staphylococcus aureus* (MRSA) was the most reported gram-positive bacteria (20.7%). *Citrobacter* (0.7%) and *Staphylococcus aureus* (10.3%) were the least reported gram-negative and gram-positive groups, respectively.

**Table 2:** Distribution of microorganisms isolated from diabetic foot ulcers. ESBL: extended-spectrum beta-lactamase

Microorganism	Number (%)
*Escherichia coli* (ESBL)	21 (14.5)
*Escherichia coli* (cepholosporinase producer)	9 (6.2)
*Pseudomonas* spp.	23 (15.9)
*Enterobacter* spp.	8 (5.5)
*Proteus mirabilis*	8 (5.5)
*Serratia marcescens*	4 (2.8)
*Klebsiella oxytoca*	13 (9.0)
*Proteus vulgaris*	6 (4.1)
*Providencia *(ESBL)	3 (2.1)
*Citrobacter*	1 (0.7)
*Candida*	4 (2.8)
*Staphylococcus aureus*	15 (10.3)
Methicillin-resistant *Staphylococcus aureus*	30 (20.7)

## Discussion

One of the most reported and crucial complications of DM is a foot ulcer. The factors that often contribute to this condition include PAD, deformities of the foot, diabetic neuropathy, unrecognized trauma, and elevated peak plantar pressure [13]. The etiology of diabetic foot ulcers is rarely an infection; however, these ulcers are prone to infection once the deformity sets in. The ulcers may not receive proper treatment with antibiotics because of the inadequate understanding of the causative organism of the condition and their pattern of sensitivity [14,15].

In our study, 146 (73%) of the wound cultures were positive for microbial growth. The microbes were 13 varieties of bacteria and one type of fungus. (Table 2) Other studies have reported higher isolate percentages [16-18]. Previous studies in Pakistan have identified gender, long-standing diabetes, cigarette smoking, and coronary artery diseases as risk factors for diabetic foot ulcers [19,20]. A study in conducted in Germany in 2008 also reported the same risk factors [21]. Moreover, sociocultural factors such as walking barefoot, inadequate care for diabetic patients, and a lower literacy rate have also been implicated as risk factors for diabetic foot ulcers [22-24]. A higher degree of risk factors for diabetic foot ulceration in Pakistan demands well-judged and far-sighted prevention and management strategies in diabetic patients.

A comprehensive profile of the causative organisms of diabetic foot ulcers is presented in this study. Gram-negative bacteria (66.2%) were more prevalent than gram-positive bacteria (33.8%). The predominant gram-negative pathogens were *Pseudomonas *spp. (15.9%) and ESBL-producing *E. coli *(14.5%) which are consistent with studies by Shanmugam et al. [25], Pappu et al. [26], and Zubair et al. [27] who reported 65.1%, 76%, and 56% gram-negative organisms, respectively, and *Pseudomonas* spp. and *E. coli* as the most common isolate. On the other hand, *S. aureus* was reported as the most common isolate by Alavi et al. [28] and Citron et al. [29] who also reported that *E. coli *was the second highest producer of ESBL.

Penicillins have been found to be the least effective in treating ulcers. This is in accordance with previous studies which reported a 100% resistance to ampicillin and 83.3% resistance to piperacillin [30]. Other studies have reported both contradicting and similar results with respect to the sensitivity of penicillins [25,30]. In our study, amikacin was the most effective antimicrobial agent, whereas Ozar et al. reported imipenem as the most effective, followed by amikacin and meropenem [30]. These results show a strong correspondence to those of our study. We also found that vancomycin and linezolid were equally effective in treating diabetic foot ulcers.

Keeping in view the results of our study, the occurrence of multi-resistant bacterial strains also foretells the possibility of longer hospital stays for patients as the process of healing may be compromised when bacteria are highly resistant to antimicrobials. The prevalence of both *Pseudomonas *spp. isolates and MRSA. Manual minimum inhibitory concentration (MIC) was not assessed as it would be tedious for all ESBL-producing clinical isolates of our study and would also be time-consuming.

Limitations of the study

This study was conducted in a tertiary care hospital in Peshawar which is in the northern part of Pakistan. The results may not be applicable to primary care hospitals, and the bacterial profile, common complications, and antibiotic resistance may be different in other parts of the country; hence, further studies should be conducted on a larger sample size to compare and evaluate the findings on a national level.

## Conclusions

Diabetic foot ulcers are caused by both gram-negative and gram-positive bacteria, and our study puts forth the view that the distribution of gram-negative bacteria is higher. Amikacin was found to be the most effective in treating the condition. The antibiogram results of our study conclude that the pathogens show sensitivity to many of the routinely used medications. However, resistance is being developed to some of the antibiotics such as trimethoprim. Therefore, the culture of the specimen to identify the causative agent and adequate knowledge of the susceptibility pattern are critical for the appropriate management of diabetic foot ulcers.
